# Specific Features of Stromal Cells Isolated from the Two Layers of Subcutaneous Adipose Tissue: Roles of Their Secretion on Angiogenesis and Neurogenesis

**DOI:** 10.3390/jcm12134214

**Published:** 2023-06-22

**Authors:** Jérôme Laloze, Marie Lacoste, Faris Marouf, Gilles Carpentier, Laetitia Vignaud, Benoit Chaput, Audrey Varin, Alexis Desmoulière, Amandine Rovini

**Affiliations:** 1 NeurIT Neuropathies Périphériques et Innovations Thérapeutiques UR 20218, Faculties of Medicine and Pharmacy, University of Limoges, 87000 Limoges, France; marie.lacoste.contact@gmail.com (M.L.); laetitia.vignaud@unilim.fr (L.V.); alexis.desmouliere@unilim.fr (A.D.); amandine.rovini@unilim.fr (A.R.); 2 Department of Maxillo-Facial, Plastic and Reconstructive Surgery, CHU Dupuytren, 87000 Limoges, France; 3 INSERM UMR 1302, Immunology and New Concepts in ImmunoTherapy, INCIT, Nantes University, 44035 Nantes, France; faris.marouf@chu-nantes.fr; 4 Gly-CRRET Research Unit 4397, Paris-Est Créteil University, 94000 Créteil, France; carpentier@u-pec.fr; 5 RESTORE Research Center, Team 2 FLAMES, Toulouse P. Sabatier University, INSERM, CNRS, EFS, ENVT, 31062 Toulouse, France; benoitchaput31@gmail.com (B.C.); audrey.varin@free.fr (A.V.); 6 Department of Plastic and Reconstructive Surgery, Toulouse University Hospital, 31100 Toulouse, France

**Keywords:** adipose-tissue-derived-stromal cells, exosomes, paracrine secretion, angiogenesis, sensory neurons, skin regeneration

## Abstract

Human-adipose-tissue-derived mesenchymal stromal cells (AD-MSCs) are currently being tested as autologous-cell-based therapies for use in tissue healing and regeneration. Recent studies have also demonstrated that AD-MSC-derived exosomes contribute to tissue repair and peripheral nerve regeneration. Subcutaneous abdominal adipose tissue (AAT) is divided into two layers: the superficial layer (sAAT) and the deep layer (dAAT). However, it is unclear whether there are particular characteristics of each layer in terms of AD-MSC regenerative potential. Using AD-MSCs purified and characterized from three abdominoplasties, we compared their secretomes and exosome functions to identify which layer may be most suitable as a source for cell therapy. Phenotypical analysis of the AD-MSCs containing stromal vascular fraction did not reveal any difference between the two layers. The AD-MSC secretomes showed a very similar pattern of cytokine content and both layers were able to release exosomes with identical characteristics. However, compared to the secretome, the released exosomes showed better biological properties. Interestingly, dAAT exosomes appeared to be more effective on neuromodulation, whereas neither sAAT nor dAAT-derived exosomes had significant effects on endothelial function. It thus appears that AD-MSC-derived exosomes from the two abdominal adipose tissue layers possess different features for cell therapy.

## 1. Introduction

Mesenchymal stem/stromal cells (MSCs) have now been widely studied over several decades [1]. They were first described in the bone marrow in 1976 by Friedenstein, then identified in other tissues such as the umbilical cord, placenta, and, more recently, in the adipose tissue (AT) [2]. Because MSCs are multipotent stem cells able to self-renew and to differentiate into many cell types including adipocytes, osteoblasts, and chondrocytes, they represent a promising cell-based strategy to treat several diseases. Adipose tissue is mainly composed of adipocytes and the stromal vascular fraction (SVF), which consists of a heterogeneous population of cells that includes adipose-derived stem cells, endothelial cells, endothelial progenitor cells, pericytes, T cells, and other immune cells including M2 macrophages [3,4].

AD-MSCs are characterized by their ability to adhere to plastic cell cultures, with a fibroblastic shape as well as possessing a specific molecular profile (CD73, CD105, CD90, etc.). These stem cells have been increasingly studied in cell therapy and regenerative medicine. They show immunomodulatory capacities [5] and present therapeutic potential for treating inflammatory diseases such as Crohn’s disease, disseminated lupus erythematosus, and scleroderma/systemic sclerosis [6,7]. Moreover, AD-MSCs can be obtained relatively easily and in large amounts from AT with minimal morbidity through lipoaspiration or abdominoplasty [8], two interventions widely used in plastic surgery, making them a major alternative source of MSCs.

The human body has various types of AT, which exhibit functional differences based on their regional distribution.

Anatomically, it is accepted that AT can be categorized into subcutaneous (deep and superficial) and internal (intrathoracic, visceral, and retroperitoneal) AT [9]. Subcutaneous AT is itself separated into two layers (superficial and deep) by the fascia superficialis, a very fine ubiquitous collagenous membrane well known to plastic surgeons. This reveals a complex architecture of the AT in which a superficial fascial system (SFS) separates the subcutaneous AT into two layers: the areolar layer or superficial AT (sAAT) and the lamellar layer or deep AT (dAAT) [10,11,12,13,14,15]. The sAAT is localized immediately below the skin and is formed by small fat lobules tightly packed between fibrous septae, derived from the SFS and oriented perpendicularly to the skin. The dAAT lies below the fascia superficialis and consists of large fat lobules, loosely packed within widely spaced vertical and oblique fibrous septae. The sAAT is widely found all over the body, whereas dAAT is more localized in specific body areas of the abdomen and hips [16,17,18,19,20,21,22].

Several studies have already compared the characteristics of AD-MSCs between the different types of AT (subcutaneous vs. internal), or indeed between different anatomical locations. These ATs have been shown to not only display morphological differences [23] but also exhibit some slight differences in their AD-MSCs depending on their location of origin [24,25]. The harvesting site is believed to affect both the yield of isolated cells as well as their growth properties [26]. However, no study to date has clearly compared the biological properties of AD-MSCs isolated from the sAAT with those from the dAAT.

In addition, the AT is considered one of the largest endocrine organs in the body, releasing secretory factors that exert systemic functions on tissues and organs. It is known that the therapeutic power of stromal cells lies primarily in their paracrine secretions that include cytokines and extracellular vesicles (EVs). EVs are small vesicles, characterized by a phospholipid bilayer, which contain a wide variety of proteins, DNA, mRNA, and miRNA molecules. There are different types of EVs that vary in origin, and in physical (size, density, sedimentation) and biochemical characteristics. Two main types of EVs have been described according to their origins: the first are small EVs termed “exosomes”, with a diameter between 30 and 150 nm, of endosomal origin, secreted after fusion with the plasma membrane of a multivesicular endosome, and which may be a promising alternative to cell therapy in regenerative medicine thanks to their relative safety (non-tumorigenic, non-immunogenic, non-infectious, non-thrombotic, or embolic); the second are microvesicles (also called microparticles) formed from budding of the plasma membrane (100–1000 nm) [27].

The use of exosomes is both more convenient and economical for clinical applications, as invasive cell collection procedures are limited. In addition, the efficiency of using exosomes has been shown in the literature [28]. In addition, microvesicles can be mass-produced using custom cell lines under controlled laboratory conditions. The long-term repetitive administration of exosomes does not cause toxicity [29]. Modification of the key biological processes is possible in order to better target the desired effects on cells [30].

Several studies have been conducted using exosomes derived from AD-MSCs, particularly in skin regeneration [31]. These have shown the role of exosomes in stimulating cell migration, proliferation, and collagen synthesis in fibroblasts, ultimately leading to accelerated healing in vivo.

In the present study, we aimed (1) to investigate, in a comparative and more exhaustive manner, AD-MSCs from the two layers of the subcutaneous AT (sAAT and dAAT) in order to optimize their use in regenerative medicine and (2) to focus on the effects of their paracrine secretions (either the secretion products in the culture supernatant or the exosomes themselves) on endothelial function and neuron outgrowth with the aim of comparing the two layers of the AT. Highlighting potential differences in AD-MSC properties between the sAAT and the dAAT would, we hope, allow the clinical practice to be modified, with the aspiration of preferably a single layer according to clinical needs.

## 2. Materials and Methods

### 2.1. Isolation, Purification, and Characterization of Human AD-MSCs

Abdominoplasties were performed in the plastic and reconstructive surgery department at the Limoges University Hospital Dupuytren with a standardized protocol and by the same operator. This study was approved by the hospital ethics committee and all patients gave oral and written consent. The lipoaspirate was washed with phosphate-buffered saline (PBS) and digested with 0.25% collagenase I (Gibco™, Grand Island, NY, USA). The obtained mixture was then filtered through nylon membranes (0.2 µm). Separation of the two layers of the AT from the fascia superficialis was performed using a scalpel and scissors and at least 10 g from each layer was placed in 50 mL tubes. To recover the SVF, the AT was digested in two steps: mechanical dissociation (Mayo scissors) followed by enzymatic digestion (type I collagenase; Gibco™) in 1X PBS on a shaker (maximum tilt and speed) for 1 h at 37 °C. Enzymatic digestion was stopped by adding complete medium (DMEM GlutaMax (1 g/L D-Glucose), 1% penicillin/streptomycin, 10% fetal bovine serum, and 20 ng/mL of basic fibroblast growth factor. The tissue remnants were filtered through a 100 μm cell filter (Steriflip, Darmstadt, Germany) to remove collagen debris and to obtain a homogeneous suspension. The filtrate was then centrifuged for 10 min at 590× *g*. The supernatant, which corresponds to the adipocytes and serum, was aspirated, and the pellet (SVF) was resuspended in complete medium or PBS and further purified by removal of residual red blood cells using the standard Ficoll-Paque (Cytiva, Marlborough, MA, USA) gradient centrifugation. AD-MSCs recovered from superficial and deep subcutaneous AT were plated at 4000 cells/cm^2^ on day 0 (passage 0 or P0) in alpha-MEM supplemented with 2% pooled human platelet lysate (Pan Biotech, Aidenbach, Germany), 1% Glutamax, 1% non-essential amino acids (Gibco) and maintained at 37 °C and 5% CO_2_. Cells were monitored daily with media renewal every 2 or 3 days and routinely counted using Trypan Blue. In addition, the AD-MSC immunophenotype was evaluated by flow cytometry using specific surface markers. The expression of positive (CD73, CD105, CD90) and negative (CD34, CD45, CD14, and CD19) markers was analyzed (MSC Phenotyping Kit, human, Miltenyi). Cell lines from three donors were used in the study.

A cell proliferation assay to characterize AD-MSC growth, by calculating the population doubling time (PDT), was then performed. The PDT (time during which the cell population doubled in number) was calculated according to the equation: PDT = culture time/PDN. The population doubling number (PDN) was calculated according to the formula PDN = log N/N0 × 3.31, where N is the number of cells at the end of the culture period and N0 is the number of cells at the start of culture. Cells were grown in 6-well plates at 4000 cells/cm^2^ at P0 and 2000 cells/cm^2^ at P1 until they reached confluence. Cells were then detached with trypsin and counted with Trypan blue, and these data were used to calculate the PDT. Finally, a comparison of the results between passages was performed.

### 2.2. AD-MSC Secretome Collection and Exosome Isolation

The AD-MSC secretome was obtained from AD-MSCs at P1–5. In order to collect the secretome, AD-MSCs at 70–80% of confluence were washed twice with 1X PBS and incubated in serum-free medium for 48 h. The supernatant was removed, pooled, and centrifuged at 300× *g* for 10 min at 4 °C to remove cell debris, and then again at 2000× *g* for 10 min at 4 °C and filtered through a 0.22 μm filter, hereafter referred to as the ‘total secretome’. The total secretome was either applied freshly or stored at −20 °C for further use. Exosomes were isolated from the total secretome by ultracentrifugation at 120,000× *g* for 2 h at 4 °C, washed in PBS, and re-centrifuged at 120,000× *g* for 2 h at 4 °C.

### 2.3. Characterization of AD-MSC Exosomes

For each exosome preparation, the concentration of total proteins was quantified with the micro-BCA assay (Thermo Fisher Scientific, Waltham, MA, USA) according to the manufacturer’s instructions. Exosomes were then resuspended in an appropriate volume of PBS or medium for further analysis or use. The concentration and size of purified exosomes were assessed by nanoparticle tracking analysis (NTA) on a Nanosight NS300 instrument. Further characterization of the isolated exosomes included labeling with antibodies against tetraspanin membrane molecules including CD9 (D3H4P, Cell Signaling Technology, Danvers, MA, USA), CD81 (SC-166029, Santa Cruz Biotechnology, Dallas, TX, USA), and CD63 (SC-5275, Santa Cruz Biotechnology), in addition to the endosomal pathway marker TSG101 (ab207567, Abcam, Cambridge, UK) and HSC70 (SC-7298, Santa Cruz Biotechnology). The membranes were probed with appropriate HRP-conjugated secondary antibodies (ab205718 and ab205719) and the signal detected by chemiluminescence was imaged on a Chemi Doc XRS (Bio-Rad, Hercules, CA, USA). The intensity of dots was analyzed using the Protein Array Analysis plugin on ImageJ.

### 2.4. Cytokine Secretion Profiling from Total Secretome and Exosomes

The cell-derived total secretome or exosomes were evaluated by the detection of human proteins including cytokines, growth factors, proteases, and soluble receptors, using the Human Cytokines Array C6 and C5 (RayBiotech, Peachtree Corners, GA, USA), according to the manufacturer’s instructions. Briefly, 1 mL of cell culture supernatant or lysed exosomes was incubated with arrayed antibody membranes for 2 h at room temperature; membranes were then washed and incubated with the mixture of biotin-conjugated antibodies for another hour at room temperature. After washing, HRP-conjugated streptavidin was added to the membranes for 1 h at room temperature. The signal was developed with the detection buffer and imaged on a Chemi Doc XRS (Bio-Rad). Chemiluminescent cytokine array data were semi-quantified with Image J. The intensity of each cytokine spot was measured on the basis of gray-scale levels. The density for each cytokine was then averaged over duplicate spot signals.

### 2.5. In Vitro Wound Healing and Angiogenic Potential of AD-MSC Total Secretome and Exosomes

Human dermal microvascular endothelial cells (HDMECs; kind gift from SILAB, Brive-la-Gaillarde, France) were isolated from human abdominoplasties with regulatory approval. Cells were grown in endothelial cell growth medium (ECGM-MV) (Promocell, Heidelberg, Germany) and used for experiments no later than P6. Two assays were performed in this study to assess the angiogenic responses of HDMECs to the AD-MSC total secretome and exosomes: (1) Tube-like formation assay: 5 × 10^3^ HDMECs per well were seeded on a Geltrex™ (Gibco)-coated 15-well µ-Slide plate (Ibidi, Gräfelfing, Germany) and treated with the secretome (50%) or exosomes (10 μg/mL) for 24 h. Images were taken on a phase-contrast microscope (Leica DMi8; 4× magnification) and processed with an adapted version of the Angiogenesis Analyzer plugin for ImageJ (National Institute of Health, Bethesda, MD, USA) [32]. The following parameters were analyzed: number of junctions and total length of master segments. Junctions refer to branching points and master segments are elements delimited by 2 junctions after limbing of the network (Figure 5A(a–d)). The normalized values were averaged over 3 experiments. (2) Wound healing migration assay: 5 × 10^3^ HDMECs/well were seeded on rat tail collagen I (Gibco)-coated Incucyte^®^ Imagelock 96-well plates and left to grow until they reached 95–100% confluence [32]. A scratch was made in each well with the Incucyte Woundmaker tool, and cells were then gently washed with PBS to remove any cell debris formed after making a scratch. HDMECs were treated either with the secretome (50%) or exosomes (10 μg/mL) for 24 h and incubated at 37 °C in 5% CO_2_ in the Incucyte Live-Cell Analysis system (Sartorius, Germany). Imaging was performed every 2 h for a total duration of 24 h with a ×10 objective. The area of wound closure was calculated and analyzed using the Incucyte Scratch Wound Cell Migration software module of the ZOOM imaging system. The experiments were performed in three technical replicates for each treatment.

### 2.6. hiPSC Differentiation into Sensory Neurons (Appendix A)

The method for differentiation of human-induced pluripotent stem cells (hiPSCs) into sensory neurons (iSNs) was adapted from available protocols [33,34]. Briefly, hiPSCs (PCi-CAU, Phenocell, Grasse, France) were cultured on human-embryonic-stem-cell-qualified Geltrex™ as colonies, in StemMACS™ iPS-Brew XF (Miltenyi Biotec, Bergisch Gladbach, Germany) with daily medium changes. When hiPSCs reached approximately 60–70% confluency, they were sub-cultured using Accutase (Stemcell Technologies, Saint-Egrève, France) (day 0), and when the same confluence was achieved, neural crest differentiation (days 1–8) was initiated by switching to NDM medium (DMEM-F12, Neurobasal Medium at a 1:1 ratio, Glutamax, N2 Supplement, B27 Supplement, non-essential amino acids, and 0.1 mM β-mercaptoethanol) containing 500 nM LDN-193189 (Selleckhem, Houston, TX, USA) and 10 μM SB431542 (Sigma, Kawasaki City, Japan). The following day (day 2), the same two factors were added, together with 3 μM CHIR-99021 (Sigma). On day 3, the medium was changed to remove LDN, and on day, 4 SB was not added, but CHIR was included until day 10, with medium changes on odd days. This results in differentiation of the hiPSC cells to neural crest progenitor cells (NCPCs). On day 11 of differentiation, NCPCs were maintained in NDM medium for expansion with the addition of 10 ng/mL of FGF-2 and 10 ng/mL of epidermal growth factor (Preprotech, Cranbury, NJ, USA). Cells were then dissociated with accutase and reseeded onto 6-well plates (Greiner Bio-One, Kremsmunster, Austria), µ-slide chambers (Ibidi, Gräfelfing, Germany), or microfluidic chambers. Differentiated sensory neurons were maintained in NDM medium with neuronal growth factors ((20 ng/mL of human β-nerve growth factor (NGF), brain-derived neurotrophic factor (BDNF), neurotrophin-3, and glial-derived neurotrophic factor (GDNF); Peprotech)) and 200 μM ascorbic acid (Sigma).

### 2.7. Axonal Regeneration

iSNs on days 15–19 underwent an axonal injury model following dissociation as single cells with accutase and re-plating in a Geltrex™-coated 96-well plate. Cells were exposed to the total secretome or exosomes for 5 days with complete media change every other day and imaged on an Incucyte 10x on days 1 and 5. To quantify neurite outgrowth induced after the re-plating, a customized version of the ImageJ Angiogenesis Analyzer plugin was used and the total branching length was normalized to the analyzed area. Three technical replicates were performed on different batches of differentiated neurons.

### 2.8. Neurosphere Formation and Quantification of Axonal Outgrowth

On day 6, NCPCs were dissociated with accutase and seeded at a density of 9000 cells/well in 150 μL of NDM medium in a 96-well non-adherent V-bottom plate (Corning, Somerville, MA, USA) for neurosphere formation. The neurospheres were allowed to grow in NDM medium for 6 days until day 12, before being transferred into a Geltrex™-coated 96-well plate and treated with either NGF (20 ng/mL), the total secretome (50% of the total volume), or exosomes (10 µg/mL) and cultured for a further 5 days. The culture medium was completely refreshed every 2 days. Images of iSN neurospheres were captured using a phase-contrast microscope (Leica DMi8; 4× magnification) on days 0 and 5. For the quantification of the area and perimeter of a neurosphere, the Image J polygon tool was used to define the edge of the neurosphere through the junction of the neurites as previously described in detail in [35].

### 2.9. Data Analysis

All experiments were repeated at least three times. Results are expressed as the mean ± standard deviation (SD). Statistical analysis was performed using GraphPad Prism using one-way analysis of variance (ANOVA) or a two-tailed unpaired *t* test where appropriate. Differences were considered to be significant when *p* < 0.05.

### 2.10. Ethics Approval

This study was approved by the Ethics Committee of Limoges University Hospital Dupuytren (protocol code ProDiCet, N° 312-2019-78 on 9 December 2019). Informed oral and written consent was obtained from all the patients.

## 3. Results

### 3.1. Characteristics of the Patients and Phenotypic Analysis of AD-MSCs

Three female patients gave their oral and written consent for this study. Their characteristics were very similar, with a mean age of 52.7 years (48–59), mean body mass index of 26.64 (25.28–27.7), and average weight loss of 5 kg (0–15). Two patients underwent delayed-deep inferior epigastric perforator (DIEP) surgery (reconstructive breast surgery after mastectomy for breast cancer), whereas the other patient underwent abdominoplasty (post-bariatric surgery). Guillain–Barré syndrome was noted for the third patient.

**Figure 1:**
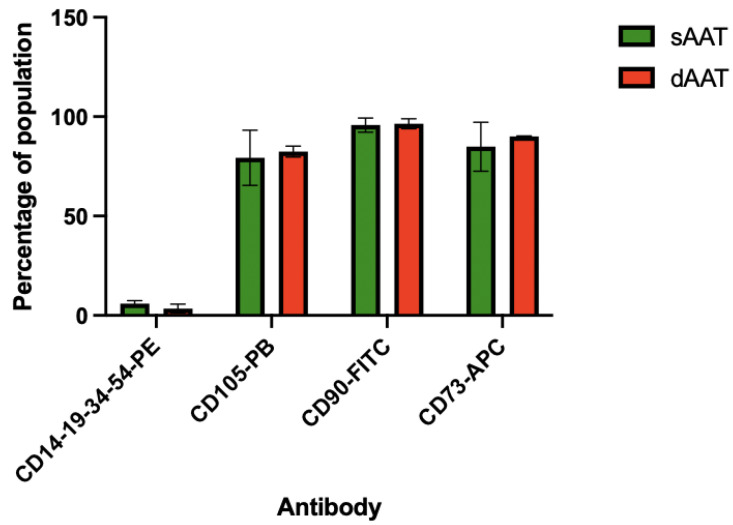
Phenotypic profiling of the extracted AD-MSCs by fluorescence-activated cell sorting at P0 (*n* = 4).

AD-MSCs from both layers of the AT were successfully isolated from the three donors. Cells showed adherence to the plastic culture flask and were characterized using flow cytometry for the expression of MSC-specific cell surface markers [36]. As shown in Figure 1, P0 AD-MSCs from both layers did not express the hematopoietic lineage markers CD14, CD19, CD34, and CD45 (3.22% for sAAT and 1.95 for dAAT). By contrast, cells from both layers were positively sorted for CD73 (89.89% vs. 93.38%, respectively, for the sAAT and the dAAT), CD105 (75.66% vs. 79.56%), and CD90 (96.97% vs. 95.24). These results confirmed the phenotypic profile of the purified AD-MSCs.

### 3.2. Proliferation Assay

**Figure 2:**
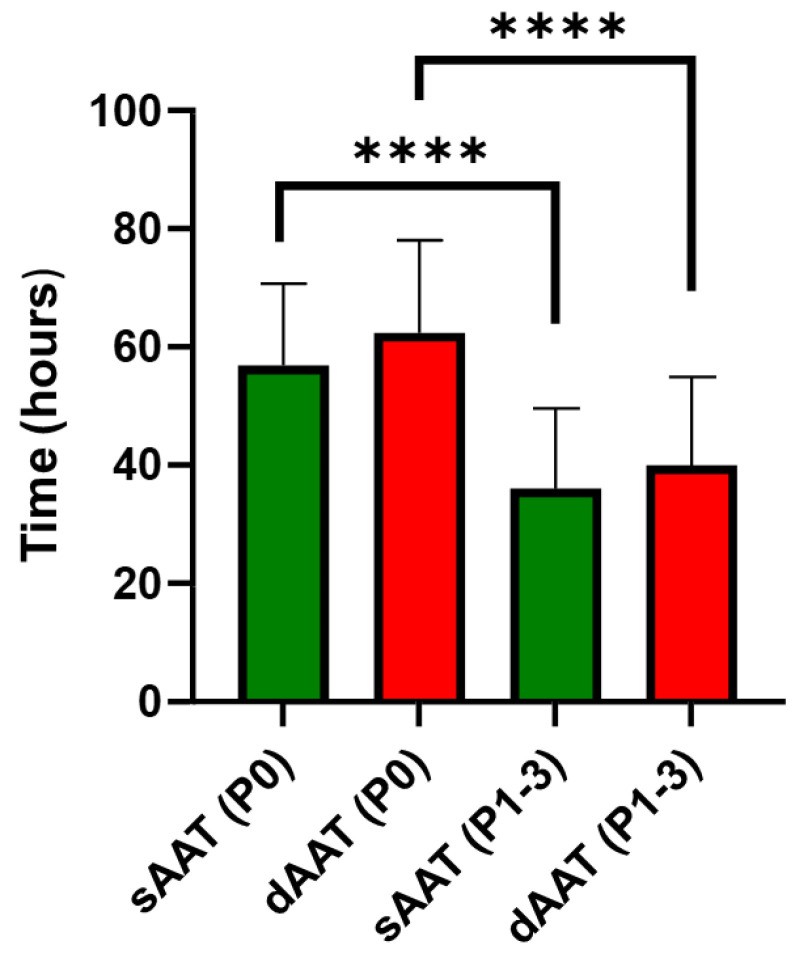
Population doubling time (hours) for AD-MSCs (superficial and deep) at passage 0 (P0) and subsequent passages from P1 to P3 (P1–3), (****, *p* < 0.0001, *n* ≥ 3).

The population doubling time (PDT) for AD-MSCs from the superficial AT layer was slightly lower than that of AD-MSCs from the deep layer, indicating a faster proliferation rate of AD-MSCs derived from the superficial layer. However, a significant decrease in PDT was observed with increased passage number for AD-MSCs from both layers. AD-MSCs derived from the superficial layer of the AT doubled their population on average over 56.89 ± 3.82 h at passage 0 (P0) and 36.04 ± 2.48 h from passage 1 to passage 3 (P1–3), whereas those derived from the deep layer of the AT at P0 and P1–3 exhibited a doubling time of 62.36 ± 3.91 h and 39.96 ± 2.73, respectively (Figure 2).

### 3.3. Cytokine Secretion Profiling from AD-MSC Total Secretome

**Figure 3:**
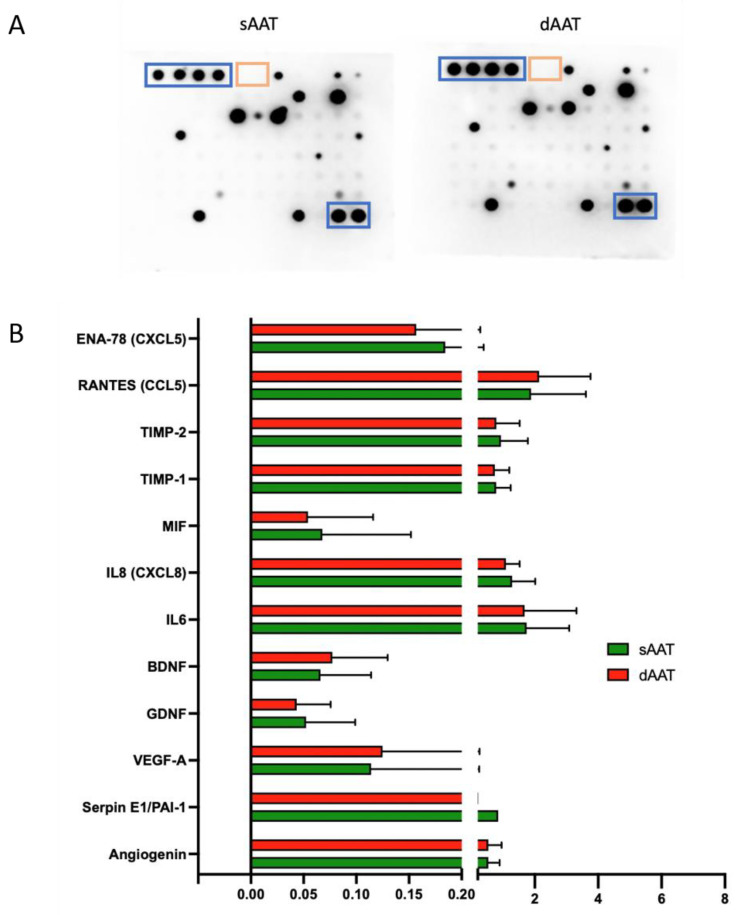
Cytokine expression profile of the conditioned media collected from AD-MSCs. (**A**) Representative image of cytokine antibody array, after incubation with the supernatant of AD-MSC DLA12 P5 from sAAT (**left**) and dAAT (**right**). Positive and negative controls are highlighted in blue and orange, respectively. Protein loading was the same for each membrane. (**B**) Quantification of the immunoblotting signals detected by chemiluminescence for the detectable cytokines in each sample (*n* = 5). T-tests showed no significant differences.

In order to define the proteins in the total AD-MSC secretome, the cell culture supernatant of P1-P6 cells from the three donors was collected following the depletion of platelet lysate for 48 h. The results shown in Figure 3A,B for the total secretome showed several cytokines at different expression levels including CCL5, IL6, angiogenin, and others. However, all detected cytokines had similar expression patterns, resulting in no significant differences being found between the two AT layers. The panel of cytokines detected was correlated with the process of wound healing and skin regeneration and more specifically with inflammation, neurostimulation, and angiogenesis. Based on these results, we then investigated the effect of AD-MSCs and their paracrine secretions on endothelial function and neuronal processes.

### 3.4. Characterization of Exosomes Secreted by AD-MSCs from sAAT and dAAT

**Figure 4:**
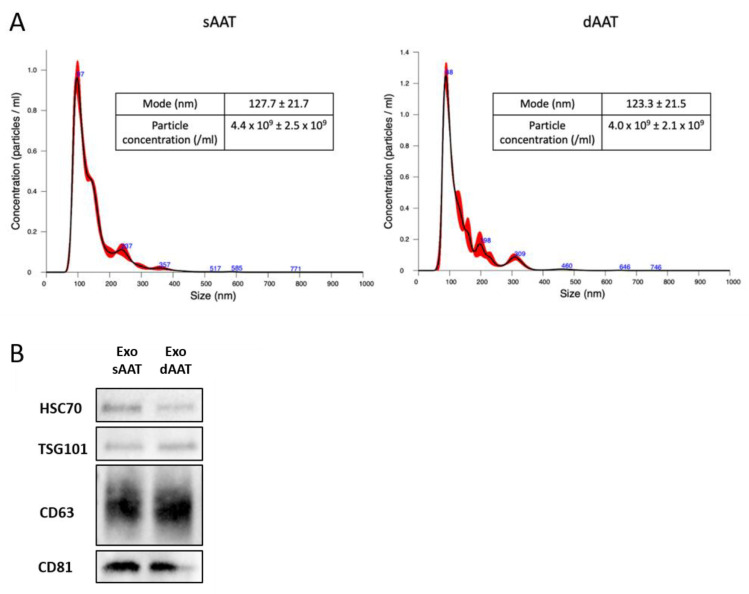
Characterization of exosomes isolated from sAAT and dAAT AD-MSCs. (**A**) Nanosight analysis of the size distribution of particles (exosomes) isolated from the culture supernatant of AD-MSCs. Particle distribution, mean, modal size, and particle concentration for the supernatant of AD-MSC sAAT (**left**) and dAAT (**right**) (*n* ≥ 3). (**B**) Western blot analysis of exosome markers. The exosomes used were derived from AD-MSCs from passage 1 to passage 3 and technical replicates were performed on AD-MSCs from the three donors (*n* = 4).

In order to determine the size distribution and the number of particles, nanoparticle tracking analysis (NTA) was performed, showing mean sizes of 167.47 nm (sAAT) and 165.4 nm (dAAT) with modes of 127.68 nm (sAAT) and 123.3 nm (dAAT) (Figure 4A), with no significant difference between exosomes in the two AT layers.

The yield of isolated exosomes from both layers, measured by NTA, also showed no significant difference. Ten milliliters of the culture supernatant of AD-MSCs from the sAAT contained 4.44 × 10^9^ particles compared to 4.02 × 10^9^ for those of AD-MSCs from dAAT. These results show that AD-MSCs derived from both layers of the AT (superficial and deep) secreted an equivalent amount of exosomes during the same period (48 h).

Western blots confirmed that the isolated nanoparticles corresponded to exosomes. The presence of the exosomal markers CD63, CD9, CD81, TSG101, and HSC70 was assessed on purified exosomes lysed with RIPA (Figure 4B). Western blot analysis demonstrated that exosomes secreted by AD-MSCs from both layers expressed CD9, CD63, CD81, TSG101, and HSC70, which are well-accepted exosome biomarkers [37,38].

### 3.5. Angiogenic and Migration Potential of HDMECs Exposed to AD-MSC Exosomes from sAAT and dAAT

**Figure 5:**
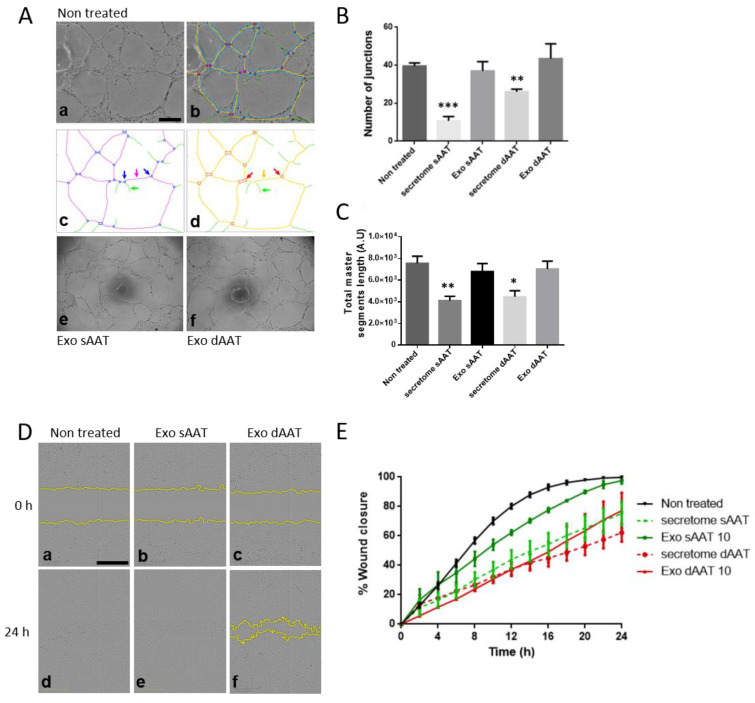
Analysis of angiogenic and migration potential of human endothelial cells in response to AD-MSCs exosomes. (**A**–**C**) HDMECs were plated on Geltrex^®^ and cultured with sAAT- or dAAT-derived exosomes (10 µg/mL) or the AD-MSC total secretome. Representative pictures of tube formation were taken after 24 h and the network properties were quantified using the Angiogenesis Analyzer ImageJ plugin. (**a**) Initial image sample of non-treated HDMECs. (**b**) Network analysis with customized version of «Angiogenesis Analyzer». (**c**) Quantified vectorial objects: green arrow, «branch»; magenta arrow, «segment»; and blue arrows, «junctions». (**d**) Red arrows, «master junctions»; orange arrow, «master segment»; and green arrow, branch. (**e**,**f**) Network formation following exposure to exosomes from sAAT and dAAT, respectively. Tube formation ability was quantified by measuring (**B**) the number of junctions and (**C**) the total length of master segments over three experiments. Two-sample T-tests were performed against the untreated control. Data are presented as mean ± standard deviation (SD). * *p* < 0.05, ** *p* < 0.01, *** *p* < 0.001. The unit of area and length is pixel (px). Scale bars, 500 µm. (**D**,**E**) The cell migration of HDMECs was measured using the wound healing assay (scratch assay). Once cells had reached 80% confluence in a 96-well plate, a scratch was made using the Sartorius wound maker tool, rinsed with PBS, and exposed to (**D**(**a**,**d**)) regular HDMEC media or (**D**(**b**,**c**,**e**,**f**)) exosomes or the secretome from either sAAT or dAAT, and plates were immediately placed in the Incucyte^®^ biostation. Images were acquired with a 10× objective over 24 h. (**D**) Representative images of HDMECs at 0 and 24 h post scratch. Scale bars 500 µm. (**E**) Quantitative analysis of the relative scratch area over time obtained using Sartorius Live-Cell Analysis system.

AD-MSC exosomes have been previously reported to promote angiogenesis [39] and wound healing [40]. To compare more specifically the role of exosomes and other released factors derived from both AT layers on the angiogenic and migratory activity of endothelial cells, we used HDMECs isolated from abdominoplasties. Low-passage-number HDMECs were treated with 10 μg/mL of sAAT- or dAAT-derived exosomes or with 50% (*v*/*v*) of the respective AD-MSC total secretome, then in vitro tubulogenesis and migration experiments were performed. When plated on Geltrex^®^ within 4–6 h, HDMECs spontaneously initiated vascular morphogenesis and formed multicellular tubular networks that were stable for up to 24 h (see Figure 5). HDMECs exposed to both sAAT and dAAT exosomes formed a complex anastomosis network (Figure 5A(e,f)), which was as extensive and well connected as the non-treated control (no significant difference found). By contrast, when half of the HDMEC media was replaced by the total secretome collected from either sAAT or dATT AD-MSCs, a marked decrease in the number of junctions and total length of the established network was observed (Figure 5B,C). The angiogenesis network formed by HDMECs exposed to purified exosomes, either from sAAT or dAAT, showed a quantitatively more complex organization than when HDMECs were exposed to the AD-MSC total secretome in terms of numbers of junctions and length of total master segments. The process of angiogenesis involves the regulation of cell survival, proliferation, differentiation, and migration, all of which are processes known to be affected by exosomes. For the scratch wound assay, cell migration was observed over 24 h (Figure 5C,D). This showed that HDMECs treated with exosomes from the sAAT were as efficient as non-treated cells in healing the scratch wound. In addition, dAAT exosomes and the total secretome from both AT layers did not allow complete wound closure.

Overall, these results suggest that AD-MSCs exosomes at a concentration of 10 µg/mL do not exert any negative biological activity on endothelial function. We can also conclude that exosomes seem to be of more interest than the secretome that inhibited angiogenesis. These data also point to there being no difference between the two layers of the AT.

### 3.6. Neurotrophic Effects of AD-MSC Exosomes from sAAT and dAAT

**Figure 6:**
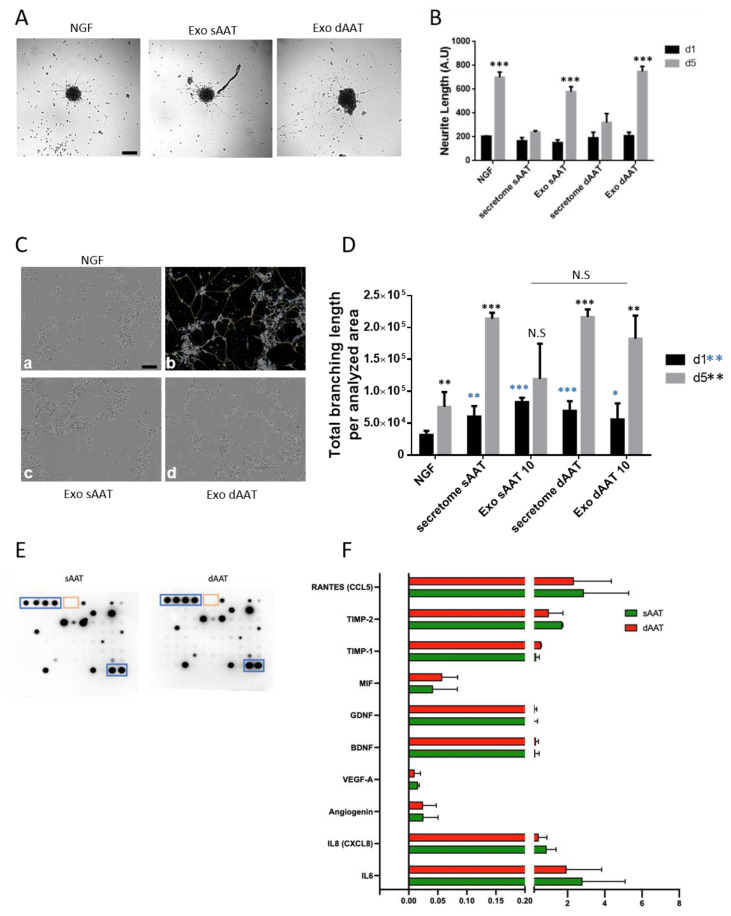
Neuroprotective properties of AD-MSCs exosome content. Assessment of neurite outgrowth (**A**,**B**) in human nociceptors grown as neurospheres and exposed for 5 days to either NGF, sAAT, and dAAT exosomes (10 µg/mL) or the total secretome. Phase contrast images of the spheres were taken (**A**) and neurite length was measured on day 1 and 5 post treatment (**B**). Scale bar, 150 µm. Restoration of neurite outgrowth (**C**,**D**) in human sensory neurons dissociated as single cells, and re-plated and exposed for 5 days to either NGF, sAAT, and dAAT exosomes (10 µg/mL) or the total secretome. (**C**) Representative pictures of the neuronal network were taken on day 1 and 5 and the network properties were quantified using a customized version of the Angiogenesis Analyzer ImageJ plugin. Scale bar, 100 µm. (**a**) Initial image sample of NGF-treated human sensory neurons. (**b**) Network analysis with customized version of «Angiogenesis Analyzer» and vectorial objects as defined in (**A**), and (**c**,**d**) neuronal network developed in response to sAAT and dAAT exosomes, respectively. (**D**) Quantification of the total branching length developed and normalized to the analyzed area. * shows significance versus the control group (NGF) on day 1 and * on day 5, * *p* < 0.05, ** *p* < 0.01, and *** *p* < 0.001. (**E**) Cytokine antibody array membranes of sAAT and dAAT exosome content with positive (blue square) and negative (yellow squares) internal controls. An equal amount of exosomal proteins was loaded for each membrane. (**F**) Quantification of the antibody array using ImageJ software.

AD-MSCs are able to secrete various pro-neurogenic cytokines to enhance neuronal growth [41]. Since cutaneous innervation plays a known role in wound healing and skin repair, we investigated the effect of AD-MSCs exosomes and the AD-MSCs total secretome on human neurite outgrowth in physiological conditions as well as in a surrogate model of nerve damage. iSNs were obtained by sequential differentiation of hiPSC into neural crest cells and further as mature neurons expressing markers of the sensory lineage and able to release neuropeptide (Appendix A). We first assessed the effect of AD-MSCs exosomes and the total secretome on iSN neurite outgrowth. Neural crest cells were differentiated as 3D neurospheres and further replated on Geltrex-coated plates to allow for neurite outgrowth in response to NGF (positive control condition), 10 µg/mL of exosomes, or the total secretome from both AT layers. Cells were grown in these conditions for 5 days with images being taken on days 1 and 5. As shown in Figure 6, neurite processes already extended 24 h post plating on Geltrex; yet, at this stage, no statistical difference was found between NGF and exosomes, or the total secretome (Figure 6A,B). In contrast, on day 5, neurites had grown significantly in response to NGF (mean values ± SEM: 700.2 ± 25.58 vs. 196 ± 21.28 on day 1, *p* < 0.0001). In comparison to this control group, only dAAT exosomes showed the same efficacy in promoting neurite outgrowth (mean values ± SEM: 700.2 ± 25.58 vs. 749.0 ± 24.39, *p* = 0.24), while both secretomes and sAAT exosomes had a significantly smaller effect than NGF (mean values ± SEM: 700.2 ± 25.58 vs. 239.0 ± 7.761, *p* < 0.0001 for sAAT secretome; 320.8 ± 43.16, *p* = 0.01 for dAAT secretome, and down to 579.2 ± 30.23, *p* < 0.0001 for sAAT exosomes) (Figure 6B). Moreover, the results of this first experiment show that, similarly to NGF, only exosomes significantly increased neurite outgrowth on day 5. Conversely, the complete secretome of AD-MSCs from both layers had no effect on neurite growth on day 5. Interestingly, exosomes derived from dAAT had a significantly higher efficacy than those derived from sAAT on neurite growth on day 5 (749.0 ±24.39 vs. 579.2 ± 30.23, *p* = 0.02).

We next examined the potential of AD-MSCs exosomes to regenerate neurites following in vitro neuronal injury since cutaneous innervation has a significant impact on a proper wound repair. The ability to re-plate differentiated cells after they have extended cellular processes within a complex network enables the study of neurite regeneration and synapse assembly in cells that are post-mitotic, as the neurites are initially damaged during re-plating. This serves as a model for investigating neurite regeneration following traumatic injury (e.g., spinal cord injury) or to identify growth-promoting molecules and screening therapeutic compounds. Differentiated iSNs detached using accutase could be successfully re-plated as a dissociated neuronal culture, with neuronal projections beginning to re-extend by 24 h (Figure 6C). Using a modified version of the Angiogenesis Analyzer Plugin, we quantified the total branching length of the neurites on day 1 and 5 following re-plating and exposure to NGF, AD-MSCs exosomes, or the total secretome (Figure 6D). On day 1, compared to NGF, both AT layer secretomes and exosomes induced a significant restoration of the neurite network (in pixels, mean values ± SEM: secretome sAAT vs. NGF: 60,300 ± 9502 vs. 31,539 ± 2642, *p* = 0.0057; exosomes sAAT: 76,591 ± 6832, *p* < 0.0001; secretome dAAT: 69,529 ± 6205, *p* = 0.0002; exosomes dAAT: 56,006 ± 10,291, *p* = 0.0440). On day 5, compared to NGF, only sAAT exosomes did not significantly improve neurite regeneration (in pixels, mean values ± SEM: secretome sAAT: 213,764 ± 3858 vs. NGF: 98,256 ± 24,331, *p* = 0.0006; exosomes sAAT: 119,376 ± 24,747, *p* = 0.5597; secretome dAAT: 216,472 ± 4907, *p* = 0.0005; exosomes: dAAT 182,328 ± 18,122, *p* = 0.03344). These results suggest that when neurons are injured, AD-MSCs exosomes and even the total secretome may be more favorable to neurite regeneration than addition of NGF alone. Finally, as with neuronal outgrowth, there is a tendency for a greater effect of dAAT exosomes compared to sAAT on nerve repair, though this was not statistically significant.

To identify the exosome content that may be involved in neurite outgrowth and repair, the expression of 83 cytokines in the exosomes from both sAAT and dAAT was analyzed. The results in Figure 6E,F show the differences in expression of neurogenic and inflammation regulators between sAAT and dAAT exosomes. Interestingly, we found that exosomes from both layers have a very similar protein content, composed of neurotrophic factors, such as BDNF and GDNF, but also of factors such as RANTES that has been described to play a neurotrophic role in dorsal root ganglia sensory neuron migration and differentiation [42] and the macrophage migration inhibitory factor MIF, which is involved in neuroinflammation and also promotes outgrowth of neuronal processes [43].

These results suggest that exosomes from the AT layers have a neurotrophic content that could be beneficial to peripheral sensory neurons during regeneration.

## 4. Discussion

To our knowledge, this is the first study to systematically and comprehensively analyze the human AD-MSCs secretome and exosomes from both sAAT and dAAT. The identification of novel aspects of the physiology of AD-MSCs and their secretome is of paramount importance for clinical practice in regenerative medicine. AD-MSCs have a unique immune modulatory function that makes them suitable for cellular therapy, including repairing tissue damaged by chronic inflammation or autoimmune diseases. However, several drawbacks have limited their therapeutic potential: those related to poor engraftment efficiency, non-specific differentiation, the potential risk of tumor formation, their short half-life, and difficulty in quality control.

In this study, two surgical indications were selected to recover AT: post-bariatric surgery or breast reconstruction where an abdominal harvest is necessary (so-called DIEP). The main originality of this study lies in the comparison of cells derived from the two layers of AT, necessitating the use of fresh tissue samples rather than tissue extracted from liposuction. Although very commonly practiced in plastic surgery, the liposuction procedure does not permit the operator to distinguish between the two layers, leaving uncertainty about the nature of the tissue removed. Additionally, studies have previously reported that there was no difference in the culture and characteristics of AD-MSCs derived from several harvesting techniques (liposuction, resection, and ultrasound) [25].

In our study, only one area was harvested for stem cell collection: the abdomen. We focused on this region since previous studies have shown that the abdomen was the richest anatomical area in terms of stem cells and that there were no morphological differences in cells between areas [25,26]. For clinical purposes, the abdomen is a preferred body area for plastic surgeons both for its ease of access and the reservoir it provides. In addition, Raposio et al. showed that AD-MSCs used in cell therapy had to be derived from subcutaneous adipose tissue to be of interest as an advanced therapy medicinal product (ATMP) [44].

Although the patient sample size was quite limited, they exhibited very similar characteristics, including relatively low weight loss. Also, patient comorbidities (history of breast cancer and Guillain–Barré syndrome) were for the most part negligible and did not affect the results obtained subsequently. It would be interesting in the future to conduct a similar study on a larger panel of patients to see if these results are confirmed. In such a study, it would also be important to match the results according to characteristics that may influence AD-MSC potential and their biological properties. Indeed, it is known that high BMI and significant weight loss can alter the morphological and metabolic appearance of stem cells.

The results of immunophenotypic characterization showed no significant difference between AD-MSCs from the two layers. Both were positive for CD73, CD90, and CD105 and negative for CD14, CD19, CD34, and CD45. These results are consistent with a previously published study [45]. The results obtained by flow cytometry confirmed the AD-MSC identity of the purified cells.

It is now accepted that the main interest in the use of these stem cells lies in their paracrine secretion: that is, their secretion of cytokines and growth factors and the release of extracellular vesicles such as exosomes. Indeed, even if 98% of AD-MSCs in vitro do not express the HLA-DR complex, they do show some immunogenicity that could decrease the effects of cell therapy. Studies have also shown that these cells are rapidly eliminated by the body (by phagocytosis and urinary elimination essentially), hence the importance of paracrine effects. For these reasons, the current focus is mostly on the study of cell secretions into the supernatant and of cytokines or EVs as a way to substitute for the cells themselves.

In terms of the proteome study, AD-MSCs from both layers of the AT showed similar patterns of cytokine expression, these being mainly involved in biological process such as inflammation, neoangiogenesis, and neuromodulation. These three major functions play crucial roles in skin healing and in various other physiological and pathological processes and are consistent with the widely reported functions of AD-MSCs. Comparison of the cytokine content obtained by an antibody array between the cell supernatant and exosome content did not show significant differences between the two layers of the AT. However, we found that the expression of neuromodulation-related cytokines, such as BDNF and GDNF, was higher in the exosomes (Figure 3B and Figure 6F), which may explain their superior effects on axonal repair and neurite outgrowth. A limitation of this antibody array analysis is the presence of exosomes in the culture supernatant. It is possible that Proteome Profiler^®^ also detects the content of exosomes, making it difficult to compare the content of the supernatant with that of the exosomes. However, we believe that the lipid bilayer of exosomes is sufficiently resistant to not to be permeabilized in our protocol, with the Proteome Profiler^®^ membrane thus not showing the contents of vesicles. Indeed, it is reported in the literature that analysis of the protein content of exosomes requires the use of specific detergents, which were not employed in our study [46,47,48]. In addition, the thawing of cells and supernatants does not compromise exosome assays, with electron microscopy studies showing that EVs are still present after thawing. Conversely, there is no certainty about the effects of freezing on exosomes, and for this reason, we excluded previously frozen exosomes in this study. A future proteomic study will be performed to further investigate in more detail the Proteome Profiler^®^ results.

We then looked at the production of exosomes by AD-MSCs of both AT layers. AD-MSCs from sAAT appeared to secrete slightly more exosomes than AD-MSCs from the deep AT (Figure 4A), though this difference was not significant.

One of the main interests for the use of AD-MSCs in cell therapy is their cytokine secretion and their secretion of exosomes. Here, we compared the effects of these two types of secretions on angiogenesis and neurite outgrowth, two key processes in skin regeneration. Some publications have previously reported that exosomes derived from AD-MSCs and bone marrow-MSCs have neuroprotective and immunomodulatory properties, attenuate neuroinflammation, promote neo-vascularization, induce neurogenesis, and reduce apoptotic loss of neural cells [49,50,51,52].

In our study, we chose to use HDMECs rather than HUVECs (human umbilical vein endothelial cells), because they are widely used in the bibliography [53], and, being derived from the dermis, are therefore more relevant for the study of skin healing. The number of junctions and the total length of the investigated segments (shown in Figure 5A–C) show the completeness of a formed vascular network. The main result in this part of the study was that the total secretome had a significant deleterious effect on the formation of a vascular network. Conversely, total exosomes had no effect compared to the control. Furthermore, no difference was found between the two separate layers of the AT for either the secretome or exosomes. These results differ from reports showing that AD-MSC-derived exosomes had the ability to promote angiogenesis [52,54]. However, most of these studies employed human umbilical vein endothelial cells, which are not representative of the cutaneous microvessel endothelium. For the secretome results, this can be attributed to the fact that the HDMEC growth medium was replaced largely by the AD-MSC supernatant. Thus, there would be a lower concentration of growth factors in the AD-MSC supernatant (which is essentially AD-MSC culture medium) compared to the HDMEC culture medium. The second test on angiogenesis focused on the migration of endothelial cells after a mechanical wound or scratch test. Here, none of the conditions showed superiority to the control. However, only the sAAT condition allowed complete healing of the wound at 24 h. Moreover, we did observe that the secretome of the two layers surprisingly looked more interesting in this respect than the exosomes. Finally, the superficial layer appeared to show greater effects and be of more interest for both the secretome and exosomes, though these differences failed to reach significance. Further studies with a greater sample size may clarify this point.

Denervation of the skin has been shown to have a major role in delayed healing [55]. In our study, the choice of iSNs derived from hiPSCs had the advantage, compared to rodent sensory neurons, of being of human origin and to provide an alternative to animal experimentation. Regarding neurite outgrowth (Figure 6A,B), there is a beneficial effect observed on day 5 of all conditions, but only the exosomes had a statistically significant effect. Moreover, very interestingly, the effect for dAAT exosomes is significantly greater than for sAAT exosomes and appeared to be larger than for the NGF positive control. This could be explained by the fact that neurite outgrowth requires growth factors other than NGF, such as BDNF and GDNF, which are present in large quantities in the exosomes (Figure 6F). Finally, the effect of exosomes is also significantly greater than that of secretions for both tissue layers, and this could be explained by their protein content. Indeed, the proteins most involved in neurogenesis found using the proteome profiler are more highly expressed in exosomes. In the case of RANTES, for example, the expression was around 3 in exosomes compared to 2 in the secretome, and similar differences were seen for GDNF (1 vs. <0.1) and BNDF (1 vs. <0.05).

Our in vitro neuronal injury experiments showed that after neuronal damage, exposure to the secretome and exosomes of both layers promoted neurite growth in a highly significant manner compared to the control. Nevertheless, no significant superiority of exosomes from the dAAT over sAAT exosomes was observed. Finally, in contrast to neurite outgrowth, in this model, the effects of the secretome were more promising than those of exosomes. This difference might be explained by their cytokine profile, with greater anti-inflammatory and antioxidant components that are reported to contribute to neuronal repair.

## 5. Conclusions

This very initial study on three donors did not highlight major differences between the two layers of AT on the studied biological parameters. Direct extensions of this work could extend to the comparison of the proteome content and the effects of the full secretome and exosomes between different anatomical regions (abdominal versus thigh versus back...). Inclusion of additional samples would allow for reassessing the slight differences noted and especially to confirm if dAAT exosomes have a greater effect on neuromodulation than on neoangiogenesis. Overall, this study supports the concept that secretion products of AD-SMCs could play an important role in cellular bioengineering in the future.

## Figures and Tables

**Figure A1:**
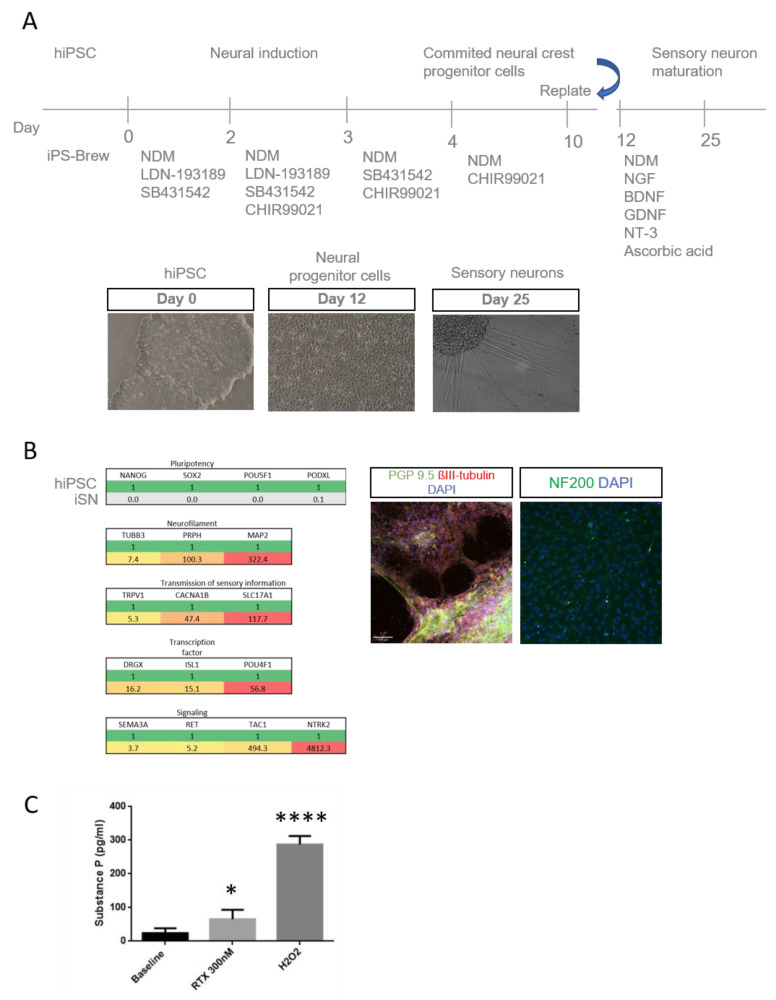
Characterization of human sensory neurons derived from hiPSC. (**A**) Differentiation timeline of iSNs with developmental steps (**top**), growth factors used at each step of differentiation (**middle**), and representative brightfield images of each step (**bottom**) are shown. (**B**) Phenotypical characterization of the iSNs populations obtained from day-12 NCPCs. The expression of markers (**left**) for the different stages was assessed by RT-qPCR comparing iSN d28 to hiPSC. Immunofluorescent (**right**) images of iSNs on day 20. For immunofluorescence, iSNs were fixed and stained for TUJ1 (red), NF200 (green), PGP 9.5 (green), and DAPI (blue). (**C**) Substance P release measured in the supernatant of iSN d25 after incubating for 45 min at 37 °C in the presence of RTX, resiniferatoxin (300 nM), or H202 (0.25%). Unpaired, two-tailed *t*-test, * *p* < 0.05, and **** *p* < 0.0001. *n* = 2–3 individual experiments from three separate sets of differentiations.

## Data Availability

The data presented in this study are available on request from the corresponding author.
